# The Prevalence of Human Papillomavirus Genotypes in Women with Precancerous Lesions and Cervical Cancer in Arequipa, Peru

**DOI:** 10.3390/life15020267

**Published:** 2025-02-10

**Authors:** Gonzalo Arturo Medina Bueno, Deyné Maribel Ticona Ramos, Claudia Amparo Mares Cuadros, Rocio Mary Quequezana Guevara

**Affiliations:** 1 Department of Obstetrics and Gynecology, Faculty of Medicine, National University of San Agustin, Arequipa 04510, Peru; dticonar@unsa.edu.pe; 2 Carlos Alberto Seguín Escobedo National Hospital of Essalud, Arequipa 04510, Peru; 3 Department of Microbiology and Pathology, Faculty of Medicine, National University of San Agustin, Arequipa 04510, Peru; cmares@unsa.edu.pe (C.A.M.C.); rquequezanag@unsa.edu.pe (R.M.Q.G.)

**Keywords:** cervical cancer, genotyping, HPV types, human papilloma virus, cervical intraepithelial neoplasia

## Abstract

The objective of this study was to determine the relationship between the prevalence of high-risk human papillomavirus (HRHPV) and age in women with cervical neoplasia or cervical cancer. This retrospective study involved 470 women referred for abnormal cervical cytology between January 2021 and December 2023. The Cobas 4800 test was used to identify HRHPV genotypes; it specifically identified genotypes 16 and 18 and grouped the other high-risk genotypes into another category. The Cobas 4800 test was performed together with colposcopy and biopsies of cervical lesions. From the analysis, we selected 470 women who underwent cervical biopsies and HPV testing. Of them, 208 (44.3%) were HPV-negative. Among the 262 women positive for HPV, 13.0% were positive for genotype 16 only, 1.3% for genotype 18 only, and 35.1% for other HPV genotypes. HPV-16 was found in 58.3% of cases of cervical intraepithelial neoplasia grade 3 (CIN 3) in women under 35 years of age and in 20.9% of cases in women over 35 years of age. Furthermore, 51.9% of patients with cervical cancer tested positive for other high-risk HPV types, whereas 30.8% had HPV-16. Although other HPV genotypes were more frequent than HPV-16 and HPV-18 in individuals with cervical cancer, HPV-16 was the most common individual high-risk genotype in women ≥ 35 years of age with CIN-3.

## 1. Introduction

Cervical cancer represents the third highest cause of cancer mortality in Peru and the sixth highest cause of cancer mortality in the American region [1]. Infiltrating cervical cancer is likely due to a combination of persistent HPV, systemic immunosuppression (HIV and immunosuppressive drugs), and a locally modified vaginal microbiota [2]. Sixteen HPV genotypes (HPV-16, -18, -31, -33, -35, -39, -45, -51, -52, -56, -58, -59, -66, -68, -73, and -82) are high-risk human papillomaviruses (HR-HPVs), suggesting an increased risk of carcinogenesis [3]. HR-HPV genotypes are found in approximately 96.4% of cervical malignancies [4]. HPV is spread through sexual contact, usually through skin–mucosal contact [5]. The most common HPV genotypes are types 16 and 18 (HPV-16 and HPV-18, respectively), which account for 25% of CIN 1 lesions, 50–90% of CIN-2 and CIN-3 lesions, and 70% of cervical carcinomas [6].

Women in their thirties are more likely to have second- or third-degree cervical intraepithelial neoplasias [7]; however, the incidence of cervical cancer is the highest in women over 48 years of age [8]. The relationship between HPV genotype and age is not fully understood: individuals under 30 years old are more likely to carry HPV types 16 and 18 [9]. The data on cervical cancer and CIN are inconsistent. HPV-16 and HPV-18 are diagnosed five years sooner than malignancies caused by other HR-HPV strains (HPV-other), but the frequency of HPV-16 declines with age [9]. Carozzi reported the same decrease in HPV-16 occurrence with advancing age in women diagnosed with cervical cancer: HPV-16 was detected in 92% of those under 35 years of age compared with 73% of those over 55 years of age (*p* = 0.036) [10].

This study aimed to determine the prevalence and distribution of high-risk HPV genotypes in women with precancerous lesions and in those with cervical cancer.

## 2. Materials and Methods

Between January 2020 and December 2023, 470 women who were referred to the Carlos Alberto Seguín National Hospital’s Gynecologic Oncology Unit in Arequipa, Peru, underwent a cervical biopsy, and HPV samples were collected. The most frequent reason for referral to the Gynecologic Oncology Service was abnormal cervical cytology.

Colposcopies were performed in controlled settings using a Zeiss colposcope. A general evaluation was performed according to the 2011 International Federation of Cervical Pathology and Colposcopy (IFCPC) classification for the cervix. This evaluation included a description of the transformation zone (TZ) as 1, 2, or 3 in relation to the visibility of the squamocolumnar junction (fully visible, partially visible, or invisible), and inflammation, bleeding, and scarring were categorized as “adequate” or “inadequate” [11].

HPV genotyping was performed using the Cobas 4800 HPV test, which is a PCR assay that detects fourteen of the high-risk HPV types. The test is used to individually identify genotypes 16 and 18, as well as a pooled group of the 12 other high-risk genotypes (31, 33, 35, 39, 45, 51, 52, 56, 58, 59, 66, and 68). HPV testing was performed according to the guidelines provided by the manufacturer. The test has a specificity of 99.4% and a sensitivity of 97.5% [12].

Every person who was referred to the unit with abnormal cytology had their cervix examined via colposcopy with 5% acetic acid. In certain cases, Lugol’s test was used to assess the cervix and identify any precancerous lesions of the vagina that were previously invisible.

At the Gynecologic Oncology Unit, a cervical biopsy was performed using Tischler biopsy forceps (Pakistan) in the area of suspicion or in the area with evidence of invasion at the Gynecologic Oncology Unit. Two or three biopsies were performed in individuals with multifocal lesions to increase the detection of high-grade dysplasia. The team that conducted this retrospective investigation included two colposcopists with different levels of experience and clinical training.

All the obtained information was entered into a database, including the colposcopic findings, cytology test results, HPV test results (if applicable), histology results, biopsy sites, transformation zone type, and epidemiologic outcomes. The histologic findings were classified as cervical carcinoma (including squamous cell carcinoma and adenocarcinoma), benign, CIN1, CIN2, and CIN3/AIS. CIN3 and AIS were combined into one group due to the rarity of glandular lesions. Age analyses were conducted for women under and over 35 years of age.

### 2.1. Statistical Analysis

The chi-square test and Yates correction were used to analyze the differences in HPV genotype prevalence between the diagnostic groups when any predicted frequency was less than 5. The significance threshold was set to 0.05. SPSS version 29 was used to perform statistical analyses (SPSS Inc., Chicago, IL, USA).

### 2.2. Ethical Considerations

The patients in both groups provided their informed consent, which included the following provisions: anonymity, secrecy, benefit, voluntary inclusion, and no information manipulation. This study was authorized by the Ethics Committee and conducted at the Carlos Alberto Seguín National Hospital’s Gynecologic Oncology Unit, an outpatient clinic of the gynecology service.

## 3. Results

**Figure 1:**
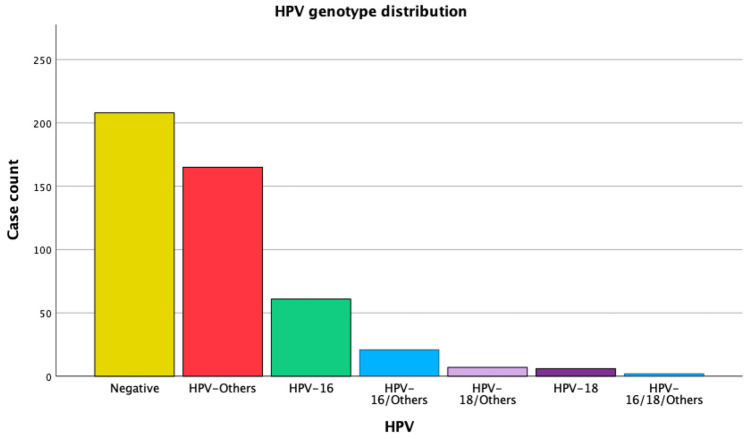
Frequency distribution of HPV genotypes.

The Gynecologic Oncology Unit performed cervical colposcopies on 600 women during the study period. No sample was taken for HPV genotyping in 130 cases because the women had a normal colposcopy or bleeding cervical tumors. As such, 470 women were included with cervical biopsy samples and HPV genotyping. The mean age of the women who were referred was 47.32 years (standard deviation ± 13.38). Twenty-eight women (44.3% of the total) had a negative HPV test. In the group of women with a positive HPV test, 13.0% were HPV-16-positive, 1.3% were HPV-18-positive, 35.1% were HPV-other-positive, and 6.4% had more than one high-risk HPV type (Figure 1 and Table 1).

Sixty-two percent of the benign lesions tested negative for HPV. Of the individuals with a diagnosis of CIN1, 41.1% had no HPV identified. HPV-other was the most prevalent result in CIN2 at 42.3%. HPV-16 was found in 29.1% of CIN3/AIS cases and in 10.9% of cases in conjunction with another HPV type. HPV-other was as prevalent as HPV-16 (29.1%) in CIN3/AIS and had a prevalence of 10.9% in cases with various HR-HPV types. The most frequent single genotype in women with cervical cancer was HPV-16 at 30.8%; however, HPV-other was found in 51.9% of individuals. HPV-18 was infrequently found, observed in only 3.8% of the women with cervical cancer. HPV-18 and HPV-other were simultaneously diagnosed in 5.8% of the cervical cancer cases. HPV was not detected in two cervical cancer patients (Table 1).

The women were divided into two age groups: those under than 35 years old and those 35 years old and older. HPV-16 prevalence was similar in the two age groups (14.0% vs. 12.7%, respectively). The HPV-18-positive rates were lower than the HPV-16-positive rates but were similar in the subgroup of women ≥35 years (1.1% vs. 1.3%). The HPV-other rates in the younger than and older than 35 years groups were approximately 44.1% and 32.9%, respectively. The incidence of coinfections was more frequent in the group of women younger than 35 years of age (12.9% vs. 4.8%) (Table 2).

The HPV-other genotypes were less prevalent in the younger age group than in the older than 34 years age group (8.3 vs. 34.9%). HPV-16 was more prevalent in CIN3 in the younger age group (58.3 vs. 32.7%). The prevalence of HPV-other was also lower in the younger age group with a CIN2 diagnosis (33.3 vs. 45%). HPV-other was more common than CIN1 in the younger age group than in the older age group (56.3 vs. 38.7%) (Table 2 and Table 3).

A total of 3 women aged <35 years and 49 women aged ≥35 years were diagnosed with cervical cancer. Among the three women with cervical cancer younger than 35 years, two had HPV-other types, and one had HPV-16/HPV-other. One woman tested positive for HPV-18, whereas eight women <35 years of age were diagnosed with HPV-16 and HPV-other. Cervical cancer in the age group ≥35 was most frequently caused by HPV-other (51.0%) and HPV-16 (32.7%). Three women in this age range had HPV-18/other, and two women with cervical cancer tested positive for HPV-18.

The 2018 FIGO classification was used to categorize the cervical cancer stages. A total of 11 of the 52 cervical cancer cases were FIGO IIIB (5 HPV-16-positive, 4 HPV-other-positive, and 2 HPV-18/other-positive); 14 were FIGO IB (4 HPV-16-positive, 9 HPV-other-positive, and 1 HPV-16/other-positive); 24 were FIGO IIB (7 HPV-16-positive, 1 HPV-18-positive, 13 HPV-other-positive, 2 HPV-16/other-positive, and 1 HPV-18/other-positive); and 1 was FIGO IIIA (HPV-18-positive). The distribution of the various HR-HPV types was equal across the various stages of cervical cancer.

## 4. Discussion

From January 2021 to December 2023, 470 patients with HPV samples obtained from cervical biopsies received treatment in our Gynecologic Oncology Unit. A total of 29.1% of the women were CIN3 HPV-16-positive and HPV-other-positive. HPV-16 was more common in individuals aged under 35 years with CIN3 than in individuals aged 35 years or older (58.3% vs. 20.9%); however, the prevalence of HPV-other was 8.3% and 34.9% among those aged ≤34 and ≥35 years, respectively. A previous study found that women with CIN2-3+ lesions younger than 30 years of age had a higher prevalence of HPV-16 and -18 than patients older than 45 years (64.3 vs. 35.1%, respectively) [7]. In that study, in the older group with CIN2-3+, the incidence of HPV-other was higher (31.9% vs. 54.6%). Similar findings were found in this study: in women with CIN-3, 34.9% of the group older than 34 years of age tested positive for HPV-other compared with 8.3% of the younger group.

In women younger than 35 years diagnosed with cervical cancer, 33.3% were HPV-16/other-positive, and 66.6% were HPV-other-positive; no diagnoses were specific to the HPV-16 genotype. Of the patients diagnosed with cervical cancer who were older than 34 years, 32.7% were HPV-16-positive, and 51.0% were HPV-other-positive. These results differ from those of Bosch et al. [13], who conducted HPV PCR testing in women with cervical cancer from 22 countries, finding HPV DNA in 93% of tumors, with 50% and 14% of these tumors being positive for the HPV-16 and HPV-18 genotypes, respectively. These results may be explained by the geographic differences in HPV distribution.

The proportion of HPV-16 infections decreased in women older than 34 years with a diagnosis of CIN3; we observed a decrease from 58.3% to 20.9% in our study cohort. Brotherton et al. found that the prevalence of HPV-16 dropped from 100% in cases with cervical cancer diagnosed between the ages of 20 and 39 years to 37.5% in women over 60 years of age [14]. In a study of Israeli women, HPV-16 and HPV-18 were found in 64.9% of cervical malignancies and in 48.2% of CIN2-3 lesions [15]. In a Chinese study of 1387 women with CIN 2-3, HPV-16 was the most frequent genotype (59.3%), followed by the HPV-58 (14.4%) and HPV-18 (6.0%) genotypes [16].

In this study, 96.2% of all cervical malignancies were positive for HPV DNA. The HPV-other group was the most common, representing 51.9% of cases. Individually, the HPV-16 genotype was the most prevalent type (30.8%), followed by HPV-18 (3.8%). These findings are comparable to those reported by Stuebs [4], who found that 96.4% of cervical cancer cases were HPV-positive; however, the HPV-16 and HPV-18 frequencies were 62.1% and 17.2% in women with carcinoma, respectively. The regional differences in HPV distribution explain this difference in the findings. Women who receive the quadrivalent HPV vaccine before age 17 have an 88% (95% CI, 0.00–0.34) lower risk of invasive cervical cancer, whereas those who receive the vaccine between ages 17 and 30 are at a 53% lower risk [17]. Women with HPV-16 are at an even higher risk of invasive cervical cancer. As a result, the risk of cervical cancer is comparatively low for vaccinated women under 34 years of age.

We observed a higher prevalence of HPV-other genotypes than HPV-16 in the group of women older than 35 years, who were at higher risk of developing cervical cancer and therefore required closer follow-up. The effectiveness of the Gardasil 9 vaccine in preventing intraepithelial neoplasias and cervical cancer caused by other HPV types not included may be reduced in our region due to the differences in HPV prevalence. The prevalence of genotype 51 is higher in vaccinated women. Furthermore, data are limited on the impact of the vaccine on cervical cancer prevalence in Peru [18]. Further studies and data on extended HPV genotyping are needed to assess the utility of the HPV vaccine.

The relationship between age and the severity of cervical intraepithelial neoplasia is important. It has been reported that the incidence and severity of CIN tend to increase with age [4]. Younger women may have higher rates of HPV infections, but these infections are transient and do not progress to CIN [2]. However, as women age, the possibility of persistent infections that evolve to CIN2-3 increases due to changes in the immune system and the accumulation of risk factors [5]. Most cases of CIN1 (60 to 70%) tend to resolve in 1 to 2 years; however, 10% of cases could progress to CIN3. Among women with CIN2, 40 to 50% regress spontaneously, and approximately 10 to 30% progress to CIN3; however, the regression rate of CIN 3 is low, being less than 10%, and it may progress to invasive cancer in 30 to 40% of women within 10 years [18].

The risk of cervical cancer due to HPV-other genotypes is extremely low in women under 35 years of age [10]. The higher prevalence of HPV-other genotypes in women with cervical cancer over 34 years of age could be associated with the competent immune system in young women detecting HPV16 and HPV18 genotypes early, thus allowing these infections to be eliminated. Although the risk of progression to cancer remains in those with persistent infection, with aging, the dynamics of the infection may change; infections by other HPV genotypes could appear and become persistent due to a decrease in the response capacity of the immune system, a longer exposure time to HPV, hormonal changes, or morbidities [17].

This study highlights the prevalence of HPV in benign lesions, CIN 1, CIN2-3, and cervical cancer. Our findings show that HPV-16 prevalence increases as the severity progresses to cervical cancer. Individuals with persistent HPV-16 are at a higher risk of developing high-grade lesions or cervical cancer and should be closely monitored. Although limiting HR-HPV testing to genotypes 16 and 18 could reduce screening costs [19], the effectiveness in detecting CIN 2+ [20] compared with broader HR-HPV tests that identify all high-risk genotypes may be reduced.

### Strengths and Limitations

The HPV status of a selected population was determined using a standardized test. The data from a therapeutically relevant cohort were reported.

This study has several limitations. First, some information was not readily available owing to the retrospective nature of this study. Second, various pathologists assessed surgical material and biopsies. Third, our study lacked control specimens, and biopsies were typically obtained from suspected lesions. The HPV test was not used by some examiners in cases of macroscopic cervical cancer, which may have impacted the final outcomes and explains the lack of cervical cancer cases. Differentiating between vaccine and non-vaccine HR-HPV genotypes is impossible with the Cobas 4800 HPV test, which could be crucial for determining cervical cancer risk in women with HPV. The lack of information on the women’s immunization status could also have impacted the findings.

The strength of this study lies in the fact that various researchers with varying specialties participated in the women’s diagnosis and treatment. Real-life data on a sizable, comparable group of women were provided.

## 5. Conclusions

Most women with CIN2-3 and cervical cancer had HPV. HPV-16 was the primary HPV genotype identified in the women with cervical cancer; however, HPV-other was more common. Women with persistent HPV-16, regardless of age, require closer follow-up because they are more likely to develop CIN2-3 and cervical cancer. Consequently, HPV genotyping is required for patients with premalignant or malignant cervical lesions. The prevalence of other HPVs was higher in older women with CIN2/3 and cancer. The frequency of HPV-16 was lower in the group of women aged 35 years or older with CIN3. Because of this, HPV screening should be age-dependent and can be adjusted by age to improve prediction outcomes.

## Figures and Tables

**Table 1:** HPV genotype distribution and cervical biopsy results.

			Cervical Biopsy					Total	*p* Value
			Benign	CIN1	CIN2	CIN3	Carcinoma		
HPV	Negative	n	128	44	19	15	2	208	Ref.
		%	62.70%	41.10%	36.50%	27.30%	3.80%	44.30%	
	HPV-16	n	14	7	8	16	16	61	<0.001
		%	6.90%	6.50%	15.40%	29.10%	30.80%	13.00%	
	HPV-18	n	1	0	1	2	2	6	<0.001
		%	0.50%	0.00%	1.90%	3.60%	3.80%	1.30%	
	HPV-other	n	53	47	22	16	27	165	<0.001
		%	26.00%	43.90%	42.30%	29.10%	51.90%	35.10%	
	HPV-16/other *	n	6	6	2	5	2	21	<0.001
		%	2.90%	5.60%	3.80%	9.10%	3.80%	4.50%	
	HPV-18/other **	n	2	2	0	0	3	7	<0.001
		%	1.00%	1.90%	0.00%	0.00%	5.80%	1.50%	
	HPV-16/18/other ***	n	0	1	0	1	0	2	0.1
		%	0.00%	0.90%	0.00%	1.80%	0.00%	0.40%	
Total		n	204	107	52	55	52	470	
		%	100.00%	100.00%	100.00%	100.00%	100.00%	100.00%	

* HPV-16/other: multiple HPV16 and other high-risk HPV infections. ** HPV-18/other: multiple HPV18 infections and other high-risk genotypes. *** HPV-16/18/other: multiple HPV16 and 18 infections and other high-risk genotypes.

**Table 2:** Distribution of HPV genotypes and cervical biopsy results in patients <35 years.

			Cervical Biopsy					Total
			Benign	CIN1	CIN2	CIN3	Carcinoma	
HPV	Negative	n	12	7	4	3	0	26
		%	35.3%	21.9%	33.3%	25.0%	0.0%	28.0%
	HPV-16	n	2	1	3	7	0	13
		%	5.9%	3.1%	25.0%	58.3%	0.0%	14.0%
	HPV-18	n	0	0	0	1	0	1
		%	0.0%	0.0%	0.0%	8.3%	0.0%	1.1%
	HPV-other	n	16	18	4	1	2	41
		%	47.1%	56.3%	33.3%	8.3%	66.7%	44.1%
	HPV-16/other	n	2	4	1	0	1	8
		%	5.9%	12.5%	8.3%	0.0%	33.3%	8.6%
	HPV-18/other	n	2	1	0	0	0	3
		%	5.9%	3.1%	0.0%	0.0%	0.0%	3.2%
	HPV-16/18/other	n	0	1	0	0	0	1
		%	0.0%	3.1%	0.0%	0.0%	0.0%	1.1%
Total		n	34	32	12	12	3	93
		%	100.0%	100.0%	100.0%	100.0%	100.0%	100.0%

**Table 3:** Distribution of HPV genotypes and cervical biopsy results in patients aged ≥35 years.

			Cervical Biopsy					Total
			Benign	CIN1	CIN2	CIN3	Carcinoma	
HPV	Negative	n	116	37	15	12	2	182
		%	68.2%	49.3%	37.5%	27.9%	4.1%	48.3%
	HPV-16	n	12	6	5	9	16	48
		%	7.1%	8.0%	12.5%	20.9%	32.7%	12.7%
	HPV-18	n	1	0	1	1	2	5
		%	0.6%	0.0%	2.5%	23%	4.1%	1.3%
	HPV-other	n	37	29	18	15	25	124
		%	21.8%	38.7%	45.0%	34.9%	51.0%	32.9%
	HPV-16/other	n	4	2	1	5	1	13
		%	2.4%	2.7%	2.5%	11.6%	2.0%	3.4%
	HPV-18/other	n	0	1	0	0	3	4
		%	0.0%	1.3%	0.0%	0.0%	6.1%	1.1%
	HPV-16/18/other	n	0	0	0	1	0	1
		%	0.0%	0.0%	0.0%	23%	0.0%	0.3%
Total		n	170	75	40	43	49	377
		%	100.0%	100.0%	100.0%	100.0%	100.0%	100.0%

## Data Availability

The data presented in this study are available upon request from the corresponding author.
